# A Comparison between Two Simulation Models for Spread of Foot-and-Mouth Disease

**DOI:** 10.1371/journal.pone.0092521

**Published:** 2014-03-25

**Authors:** Tariq Halasa, Anette Boklund, Anders Stockmarr, Claes Enøe, Lasse E. Christiansen

**Affiliations:** 1 Section of Epidemiology, The National Veterinary Institutes, Technical University of Denmark, Copenhagen, Denmark; 2 Department of Applied Mathematics and Computer Science, Technical University of Denmark, Lyngby, Denmark; Centers for Disease Control and Prevention, United States of America

## Abstract

Two widely used simulation models of foot-and-mouth disease (**FMD**) were used in order to compare the models’ predictions in term of disease spread, consequence, and the ranking of the applied control strategies, and to discuss the effect of the way disease spread is modeled on the predicted outcomes of each model. The DTU-DADS (version 0.100), and ISP (version 2.001.11) were used to simulate a hypothetical spread of FMD in Denmark. Actual herd type, movements, and location data in the period 1^st^ October 2006 and 30^th^ September 2007 was used. The models simulated the spread of FMD using 3 different control scenarios: 1) A basic scenario representing EU and Danish control strategies, 2) pre-emptive depopulation of susceptible herds within a 500 meters radius around the detected herds, and 3) suppressive vaccination of susceptible herds within a 1,000 meters radius around the detected herds. Depopulation and vaccination started 14 days following the detection of the first infected herd. Five thousand index herds were selected randomly, of which there were 1,000 cattle herds located in high density cattle areas and 1,000 in low density cattle areas, 1,000 swine herds located in high density swine areas and 1,000 in low density swine areas, and 1,000 sheep herds. Generally, DTU-DADS predicted larger, longer duration and costlier epidemics than ISP, except when epidemics started in cattle herds located in high density cattle areas. ISP supported suppressive vaccination rather than pre-emptive depopulation, while DTU-DADS was indifferent to the alternative control strategies. Nonetheless, the absolute differences between control strategies were small making the choice of control strategy during an outbreak to be most likely based on practical reasons.

## Introduction

Foot-and-mouth disease (**FMD**) is a highly contagious disease of ruminants and pigs that can cause large economic damage [1]. Several countries have imposed strict legislations and control strategies to eradicate FMD, such as the western European countries [2]. Despite of the successful eradication of FMD from these countries, some suffered severe outbreaks during the past 15 years, which indicates that FMD remains a constant threat to FMD-free countries. Following the 2001 UK outbreak, the EU has revised the regulations, in which the use of emergency vaccination was emphasized, and more emphasis on member states to show permanent awareness and preparedness to an FMD outbreak was enforced [3].

Simulation models are widely used to support veterinary authorities to setup contingency plans for FMD awareness and preparedness [1], [4], [5], [6], [7]. They are also used to study the potential spread of FMD and to evaluate potential control strategies to minimize the impact of the outbreak [7], [8]. During the 2001 UK outbreak, simulation models were used to help the veterinary authorities control the spread of the outbreak [9], [10]. Despite of the wide use of FMD simulation models, different models may substantially differ from each other due to different assumptions regarding the modeled processes. Moreover, models can differ in their flexibility to include changes to the models’ basic structure, their data requirement to run, and their ease of use. For example, the InterSpread Plus model (**ISP**) [11], [12], [13], [14] has a user friendly interface, but it is not flexible, when it comes to including changes to the basic structure of the model. On the other hand, the Davis Animal Disease Simulation model (**DADS**) that has been further developed at the Technical University of Denmark to **DTU-DADS** [15], [16] requires good programming skills, and hence is not user friendly. However, because it is possible to include changes to the model structure, this model is very flexible. In order to understand the simulated processes, the spread mechanisms and the results of the models, it is important to understand how the differences between models affect the results.

Because of the absence of outbreak data in some countries, and hence the difficulty to validate outcomes of an FMD simulation model, relative validity has been proposed [17], [18]. This method suggests that two or more scenarios are defined and two or more independently developed models are used to simulate the spread of disease using these test scenarios [18]. Agreement among the different models in their prediction provides evidence that the developers of each model were consistent in their approach to simulate the spread of the disease [18]. The spread of FMD was compared using 3 simulation models; ISP, the North American Animal Disease Spread (NAADSM) and the Australian model (AusSpread) [18]. The authors found that the predicted outcomes were statistically significantly different between the different models. Nonetheless, the authors did not provide a detailed description of the effects of differences between models on the predicted outcomes of the models.

The objective of this paper is to simulate a hypothetical spread of FMD in Denmark using two widely used simulation models of FMD spread (DADS and ISP), in order to compare the models’ predictions in term of disease spread, consequence, the ranking of the applied control strategies, and the effect of the way disease spread is modeled on the predicted outcomes of each model.

## Materials and Methods

### Data Description

Both simulation models used the same herd data, which contained information on all Danish cattle, swine, sheep and goats herds in the period from 1^st^ October 2006 until 30^th^ September 2007. For each herd, the herd data included the Danish Herd Identification System, referred to as CHR number, herd type, UTM geo-coordinates, number of animals, and number of off-farm animal movements per day. Herds were categorized into 3 categories; cattle, swine, and small ruminants (in this paper referred to as “sheep”). Cattle herds were categorized as dairy or non-dairy herds. Swine herds were categorized into 19 different types based on their production type and SPF (specific pathogen-free herd) status [19]. The number of animal movement was divided into animal movement from a herd to another and animal movement to the abattoir. For swine herds, animal movements were described as movements of either sows or weaners. When a farm included several animal species, each species was given a different ID and set as a different herd on the same location and with the same CHR number.

The input parameters of the models were based on Danish data, the literature and personal communication to experts [15]. Due to the large number of input parameters used in the models, we have described only parameters that influence the difference between the two models in this paper. All other parameters are described in a previous publication [15].

### The Simulation Study

#### General framework

A hypothetical spread of FMD between herds in Denmark was simulated using two spatial simulation models; namely DTU-DADS (version 0.100), and ISP (version 2.001.11). The DADS model (version 0.05) was upgraded to DTU-DADS [15], to incorporate changes necessary to model FMD spread in Denmark. The simulation starts with the models loading the input data, and thereafter selecting the index herd, which is the first infected and detected herd in the epidemic. The index herd was randomly chosen for each herd type and when relevant for different animal densities. The index herds were 1,000 cattle herds located in high density cattle areas and 1,000 in low density cattle areas, 1,000 swine herds located in high density swine areas and 1,000 in low density swine areas, and 1,000 sheep herds. This was done to consider the variation between index herds, and for each index herd, the epidemic was simulated only once ( = 1 iteration). The same index herds were used in both models and in all control scenarios to minimize variation between the models and scenarios.

#### Disease spread and dynamics

Spread of infection between herds was simulated through 7 spread mechanisms: 1) direct animal movement between herds; 2) abattoir trucks; 3) milk tankers; 4) veterinarians, artificial inseminators, and/or milk controllers (referred to as medium risk contacts); 5) visitors, feedstuff and/or rendering trucks (referred to as low risk contacts); 6) markets; and 7) local spread.

Based on actual animal movement data, a rate of animal movements per day was calculated for each herd. The individual daily movement rate was used as lambda in a Poisson distribution to represent the number of movements per day. Similarly, a rate of abattoir deliveries per day was calculated based on herds’ actual data and used in a Poisson distribution to simulate the number of movements to the abattoir per day from the infectious herd. Thereafter, the number of herds visited by an abattoir truck on the way to the abattoir following visit to an infected herd was estimated from a Poisson distribution with a lambda depending on the herd type. For all milking herds, the average probability of having milk picked up was used as lambda in a Poisson distribution describing contacts between herds by milk tankers. Likewise, medium and low risk contacts were simulated, but with different lambdas and risks of infection [15]. Once an infectious herd had a contact with a susceptible herd, the susceptible herd might become infected based on probabilities of infection per contact type [15]. It was assumed that all herds are equally susceptible, while the infectiousness was related to the proportion of infected animal within the herd. Because markets in Denmark are restricted to cattle only, an infection spreading from a market can initially affect only cattle herds [15]. Local spread was defined as infection of susceptible herds within a 3 km radius around the infected herd due to unexplained reasons, such as rodents, birds, flies and a limited airborne spread.

The disease was modelled to always start in one herd (the index case) and develop until the disease was detected, and hence the herd was depopulated. The period from a herd starts showing clinical signs and until detection was dependent on the herd type, e.g. cattle herds were detected faster than sheep herds, because some sheep do not show clinical signs. Moreover, herds within the protection and surveillance zones would have higher probability of detection, because of surveillance. Detection of the first infected farm was assumed to always be at day 21 following the start of the epidemic. This was based on experience from the UK [20], [21] and the Dutch 2001 FMD outbreaks [22]. In the simulations, the infection spread freely between herds during the first 21 days.

#### Basic control measures following detection of infection

After detection of the first infected herd, a set of default control strategies were applied representing the basic scenario. These included: 1) depopulation, cleaning and disinfection of detected herds; 2) a 3 days national stand still on animal movements in the country; 3) a 10 km radius zone (surveillance zone) around the detected herds; in which movements between herds and out of the zone were restricted and herds were surveyed one time before lifting the zone; 4) a 3 km radius zone (protection zone) around the detected herd, in which movements between herds and out of the zone were restricted, and herds are surveyed during the first week and a second time, 21 days later; 5) backward and forward tracing of contacts from and to detected herds. When a herd had received animals from a detected herd, the receiving herd was also depopulated and disinfected, while in case of other kind of contacts, the herd was surveyed. When a herd was subject to surveillance, the animals were inspected for clinical signs of FMD. In case of sheep herds, the animals were also sampled for serological analysis [15]. The daily animal depopulation capacity was set at 2,400 ruminants and 4,800 pigs [15]. Detected herds had higher priority for depopulation than traced herds. In case of several herds on the same farm, all herds on the farm were depopulated, when one herd was depopulated.

#### Simulated scenarios

Three spread scenarios were run in the models. The scenarios were: 1) the basic scenario, in which the EU and Danish control strategies were implemented as explained bellow, 2) pre-emptive depopulation, including the basic scenario plus depopulation of herds within 500 meters around detected herds, and 3) suppressive vaccination, including the basic scenario plus emergency vaccination of herds within 1,000 meters around detected herds. Vaccination and depopulation were initiated 14 days following the detection of the first infected herd.

When a susceptible herd was vaccinated, the herd was assumed to be susceptible for 4 days before the immunity would start to build up and reach its maximum potential at day 9 following vaccination. Vaccinated herds that became infected would be fully infectious, if they had been vaccinated ≤4 days before exposure to the virus, otherwise, the infectiousness reduced until day 9 following vaccination, where it was reduced by 90% [15]. The efficacy of the vaccine was obtained from a meta-analysis study on FMD vaccine efficacy [23].

The daily animal vaccination capacity was assumed to be 60,000 ruminants and 50,000 pigs [15]. Before vaccination, cattle and pig herds were clinically surveyed and sheep herds were serological surveyed. Thirty days following vaccination, the herds were surveyed again, before the vaccination zone was lifted.

Vaccinated herds were assumed to be depopulated after the end of the outbreak [5]. In this paper, only the effect of suppressive vaccination is presented. Results simulating the effect of protective vaccination are presented in a previous publication [15].

### Differences between the Models

Despite that the models were setup to simulate the same spread scenarios, there are several dissimilarities between the models and it was not possible to make all simulations identical in the two models. These differences are:

When direct and indirect contact is modelled, a susceptible herd can become infected, based on a distance-based probability, a probability of contact between the different herd types, and a probability of disease transmission. In the DTU-DADS, these risks are multiplied and then a herd is selected from all herds within the country. In the ISP, the model will first select a distance band and then a herd will be selected within the band. Herds of the same type within a distance band will have similar probability of selection.

Disease spread within a herd in DTU-DADS is modeled stochastically. This means that disease spread within herds of the same type can be different. In ISP, herds of the same type would have similar patterns of within herd disease spread. This means that the infectiousness of a herd can be different between DTU-DADS and ISP, despite of similar herd characteristics and time of infection.

Several parameters are stochastic in the models. However, the way the stochasticity of these parameters is implemented can be different for some of the parameters. In DTU-DADS, risk of infection following low, medium or high risk contacts are stochastic across iterations. This means the risk for a specific herd would be the same during the iteration. Such parameters are stochastic per day in ISP, which means that the values differ between days within iteration for the same herd.

When modeling the number of contacts the truck makes on its way to the abattoir to deliver pigs and sheep, ISP uses the herds-specific abattoir lambda to determine whether a movement to the abattoir will occur at that day. Thereafter, it determines the number of herds that will be contacted on the way to the abattoir, based on a probability distribution function. In DTU-DADS, the herd-specific abattoir lambda is used in a Poisson distribution to determine the number of contacts a truck makes picking up pigs or sheep on its way to the abattoir.

In DTU-DADS all infected herds will eventually be detected. However, small herds might be infected and then recover without being detected in ISP.

Other differences do exist, but are not presented here, because of their minor impact on models’ predictions, following investigation through sensitivity analysis [15].

### Cost-benefit Analysis

The costs and losses due to the epidemics per control scenario were calculated as explained previously [15]. Briefly, the total costs of an epidemic were the sum of the direct and indirect costs. The direct costs consisted of surveillance, depopulation, cleaning and disinfection, empty stable, compensation, national standstill, and vaccination costs. The indirect costs included losses incurred from restrictions on exports to EU and non-EU countries. Total costs were calculated per iteration and their summaries were thereafter estimated.

### Comparison between the Models

The predicted epidemiologic, total costs and epidemic area outputs of each scenario were compared between the two models. Epidemiologic predictors consisted of number of infected premises and the epidemic duration. The spatial spread (or epidemic area) was calculated by plotting the locations of the detected herds per iterations and constructing a minimum convex hull around the herds and then measuring the area of the resulting convex hull polygon. Predictions of each scenario of the two models were compared using the Wilcoxon Signed Rank Sum test in R 2.14.0 [24].

## Results

### Basic Scenario

**Figure 1:**
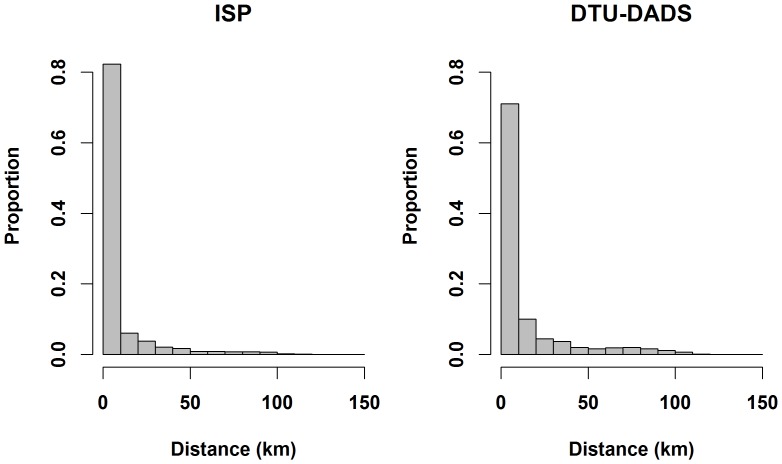
Distribution of distances (km) between infected herds and the source herd in two stochastic models simulating spread of FMD in Denmark (DTU-DADS (black) and ISP (gray)). Epidemics were initiated in cattle herds located in high density cattle area, using the basic control strategy(EU and Danish regulation of FMD control). Distances over 150 km were removed (<0.01% of distances).

When epidemics were initiated in cattle herds located in high density cattle area, the proportion of disease spread through animal movements, indirect contacts and local spread predicted by DTU-DADS were, consecutively, 0.003%, 0.547% and 0.45%, while ISP prediction of disease spread through these mechanisms were, consecutively, 0.01%, 0.33% and 0.66%. Similar trends were observed when epidemics were initiated using the other index herd types. The distributions of distance between the infected herd and the source herd from both models are shown in Figure 1. It shows that DTU-DADS tends to have higher probability to spread the disease over long distances, while ISP tends to spread the disease over shorter distances, and hence cluster the spread in smaller areas than DTU-DADS. The 5^th^, 25^th^, 50^th^, 75^th,^ 95^th^ percentiles and maximum values of the predicted distances by DTU-DATS were, respectively, 0.00, 0.55, 1.92, 13.61, 72.44 and 163 km, while the predicted values by ISP were, respectively, 0.00, 0.53, 1.47, 3.49, 43.99 and 300 km.

DTU-DADS predicted larger epidemics of longer duration than the ISP, except from epidemics starting in cattle herds located in high density cattle areas. In these epidemics, ISP predicted larger and longer duration epidemics (Table 1). There is a significant difference between the results of DTU-DADS and ISP in the basic scenarios, when the epidemic started in cattle herds located in high density cattle and swine herds located in high density swine areas and in sheep herds (Table 1). When the epidemic started in cattle herds located in low density cattle areas, the epidemiologic predictors (epidemic duration, number of infected and depopulated herds) show an insignificant difference between the models’ results (Table 1). However, the differences in total costs and epidemic area between the 2 models were significant, in which DTU-DADS predicted wider spread and more costly epidemics. Generally, DTU-DADS seems to predict disease spread over larger areas than ISP (Table 1). This is not only when DTU-DADS predicted larger number of infected herds, but also when the difference in the predicted number of affected herds was not significantly different between the 2 models, as the case when epidemics started in cattle herds located in low density cattle area (Table 1). In this case, the predicted epidemic area by DTU-DADS was almost double the size of the predicted area by ISP (Table 1). Results from epidemics started in swine herds located in high and low density swine areas were very similar, and therefore we chose to present only those started in swine herds located in high density swine areas.

**Table 1:** Epidemiological and economic results of a simulated FMD-epidemic in Denmark, using two simulation models: DTU-DADS and ISP.

Scenario and outcome parameter	Model - Median (5^th^ and 95^th^ percentiles)	
	DTU-DADS	ISP
**High cattle**		
Duration (days)	56**(16–142)	80 (5–255)
Infected	67**(13–245)	137 (3–696)
Depopulated	67**(13–245)	141 (3–718)
Total costs (€×10^6^)	565**(402–946)	665 (399–1,137)
Area (km^2^)	9,869*(567–28,687)	11,114 (0–35,178)
**Low cattle**		
Duration (days)	71 (19–179)	66 (2–226)
Infected	94 (15–371)	81 (2–521)
Depopulated	94 (15–371)	80 (1–539)
Total costs (€×10^6^)	608**(416–1,061)	547 (363–1,101)
Area (km^2^)	11,414**(339–36,207)	5,994 (0–32,588)
**High swine**		
Duration (days)	43**(8–130)	25 (2–180)
Infected	36**(5–195)	12 (1–313)
Depopulated	36**(5–195)	13 (1–322)
Total costs (€×10^6^)	498**(376–869)	429 (341–961)
Area (km^2^)	5,053**(11–27,254)	771 (0–22,680)
**Sheep**		
Duration (days)	38**(6–139)	9 (2–155)
Infected	29**(3–198)	4 (1–222)
Depopulated	29**(3–198)	4 (1–233)
Total costs (€×10^6^)	476**(364–876)	410 (345–723)
Area (km^2^)	3,881**(0–24,473)	1 (0–17,538)

**refers to a **p-value <0.01**, *refers to a **p-value <0.05,** and no sign refers to a **p-value ≥0.05.**

**Basic control measures** are simulated to control the epidemic. Epidemics are starting in cattle herds located in high and low density cattle areas, swine herds located in high density swine areas and in sheep herds, resulting in 5000 simulated epidemics. Results are given as medians (5–95%).

**Figure 2:**
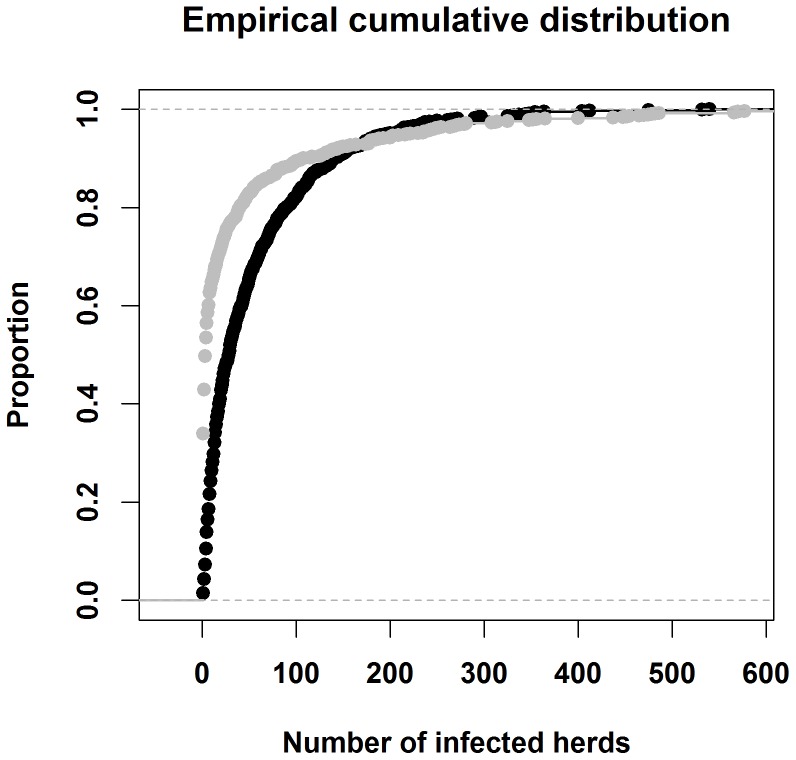
Empirical cumulative distribution of the number of infected herds predicted by the DTU-DADS (black) and ISP (gray), when the epidemic started in sheep herds and under the basic scenario, in which the EU and Danish regulation of FMD control were simulated.

ISP shows larger variability’s and extreme situations than the DTU-DADS model as presented in the 5^th^ and 95^th^ percentiles of the predicted outcomes (Table 1). When the epidemic started in sheep herds, DTU-DADS showed significantly larger number of infected herds than the ISP (Table 1). However, ISP showed larger variability and more extreme epidemics than DTU-DADS (Figure 2).

### Depopulation and Vaccination Scenarios

In the depopulation and vaccination scenarios (Tables 2 and 3), we generally observed similar trends in differences between DTU-DADS and ISP as those observed in the basic scenario. DTU-DADS showed significantly larger, wider spread and costlier epidemics than the ISP, except when the epidemic started in cattle herds located in high density cattle areas. When the epidemic started in cattle herds located in low density cattle areas using vaccination 14 days following the detection of the first infected herd, the difference between the 2 models’ prediction became significant (Table 3).

**Table 2:** Epidemiological and economic results of a simulated FMD-epidemic in Denmark, using two simulation models: DTU-DADS and ISP.

Scenario and outcome parameter	Model - Median (5^th^ and 95^th^ percentiles)	
	DTU-DADS	ISP
**High cattle**		
Duration (days)	46**(16–100)	66 (5–184)
Infected	59**(12–177)	109 (3–469)
Depopulated	84**(13–282)	175 (3–806)
Total costs (€×10^6^)	533**(403–773)	614 (398–948)
Area (km^2^)	9,372**(527–25,448)	9,779 (0–31,422)
**Low cattle**		
Duration (days)	51 (18–117)	52 (2–166)
Infected	71 (15–231)	59 (2–367)
Depopulated	104 (18–368)	93 (1–604)
Total costs (€×10^6^)	543**(412–811)	510 (363–936)
Area (km^2^)	9,672**(338–30,628)	4,608 (0–29,507)
**High swine**		
Duration (days)	37**(8–96)	23 (2–132)
Infected	31**(5–126)	11 (1–195)
Depopulated	43**(5–205)	16 (1–341)
Total costs (€×10^6^)	480**(376–731)	422 (340–805)
Area (km^2^)	4,386**(11–22,704)	642 (0–19,010)
**Sheep**		
Duration (days)	34**(6–100)	9 (2–133)
Infected	25**(3–130)	4 (1–157)
Depopulated	34**(3–210)	4 (1–285)
Total costs (€×10^6^)	464**(364–716)	405 (345–681)
Area (km^2^)	3,301**(0–19,842)	1 (0–13,396)

**refers to a **p-value <0.01**, *refers to a **p-value <0.05,** and no sign refers to a **p-value ≥0.05.**

Basic control measures **plus pre-emptive depopulation in 500 meters** are simulated to control the epidemic. Epidemics are starting in cattle herds located in high and low density cattle areas, swine herds located in high density swine areas and in sheep herds, resulting in 5000 simulated epidemics. Results are given as medians (5–95%).

**Table 3:** Epidemiological and economic results of a simulated FMD-epidemic in Denmark, using two simulation models: DTU-DADS and ISP.

Scenario and outcome parameter	Model - Median (5^th^ and 95^th^ percentiles)	
	DTU-DADS	ISP
**High cattle**		
Duration (days)	47**(16–100)	59 (5–141)
Infected	60**(12–193)	93 (3–368)
Depopulated	60**(12–193)	96 (3–383)
Vaccinated	90**(3–350)	160 (0–711)
Total costs (€×10^6^)	535**(400–788)	573 (400–803)
Area (km^2^)	10,473 (549–25,236)	8,218 (0–28,349)
**Low cattle**		
Duration (days)	52*(19–103)	48 (2–137)
Infected	74**(15–232)	53 (2–287)
Depopulated	74**(15–232)	53 (1–303)
Vaccinated	117 (7–434)	84 (0–579)
Total costs (€×10^6^)	546**(410–799)	497 (365–820)
Area (km^2^)	11,683**(351–31,036)	4,136 (0–25,236)
**High swine**		
Duration (days)	37**(8–86)	23 (2–115)
Infected	31**(5–124)	11 (1–155)
Depopulated	31**(5–125)	12 (1–161)
Vaccinated	41**(0–253)	12 (0–357)
Total costs (€×10^6^)	479**(375–694)	421 (341–728)
Area (km^2^)	6,784**(10–21,497)	627 (0–17,225)
**Sheep**		
Duration (days)	35**(6–85)	9 (2–98)
Infected	26**(3–125)	4 (1–114)
Depopulated	26**(3–125)	4 (1–118)
Vaccinated	34**(0–251)	0 (0–270)
Total costs (€×10^6^)	469**(365–691)	404 (346–598)
Area (km^2^)	5,930**(0–19,977)	1 (0–10,664)

**refers to a **p-value <0.01**, *refers to a **p-value <0.05,** and no sign refers to a **p-value ≥0.05.**

Basic control measures **plus suppressive vaccination in 1000 meters** are simulated to control the epidemic. Epidemics are starting in cattle herds located in high and low density cattle areas, swine herds located in high density swine areas and in sheep herds, resulting in 5000 simulated epidemics. Results are given as medians (5–95%).

Generally, the models showed that zone depopulation or suppressive vaccination resulted in significantly shorter epidemics, fewer infected herds and cheaper epidemics than the basic scenario in both models (p-values <0.05). Using DTU-DADS, there was no significant difference in the costs of epidemics between depopulation in 500 meters and suppressive vaccination in 1 km control scenarios (p-values >0.05), regardless the index-herd type. However, using ISP, suppressive vaccination in 1 km was generally a cheaper choice than depopulation in 500 meters (p-value <0.05). Nonetheless, given the large variation, the difference in the absolute values is rather small.

## Discussion

Generally, DTU-DADS showed significantly larger and longer duration of outbreaks than the ISP, when the epidemic started in swine herds located in high density swine areas and in sheep herds. The opposite was true when the epidemic started in cattle herds located in high density cattle areas. When epidemics started in cattle herds located in low density cattle areas, there was no significant difference in the predicted number of infected herds and epidemic duration between the 2 models, but DTU-DADS predicted larger epidemic area and costs than ISP. The tendency that DTU-DADS predicts larger epidemics and epidemic area than ISP could be explained by the way the newly infected herds are selected. In DTU-DADS, when an infected herd would infect other herds, the newly infected herds would be selected from all herds in the country based on distance and contact probabilities. In ISP, a distance band is drawn around the infected herd and then newly infected herds are selected from within the band. Herds of the same type would have similar probability of infection. In case no herds with positive probability of infection were found in the band, a new band will be selected with a maximum retries sat to 100 times in the current project. This means that in DTU-DADS, herds located close to and herds located far away from the infectious herd would be subjected to selection directly in one step. These herds would of course have different probabilities of selection, in which herds located far away would have lower probabilities than herds located close by. Nonetheless, the number of susceptible herds located far away is very large, which means that some might be selected more often compared to the way selection of new infected herds is carried out in ISP. This is because, in ISP such herds would have extremely small chance of selection, because the closer bands would most likely be selected. This means that DTU-DADS tend to spread the disease over longer distances and thus generally larger epidemics and epidemic area than ISP. This can be seen from Figure 1, which shows clearly that DTU-DADS have a higher chance to spread the disease over longer distances than ISP.

Furthermore, local spread dominated the different types of spread mechanisms in ISP (66%), while lower percentage (45%) of infection through local spread was predicted by DTU-DADS. This indicates as well that ISP tends to restrict outbreaks to a small area, while DTU-DADS would spread them out over longer distances, and hence larger areas. We speculate that the higher percentage of disease spread through local spread, and the short distance jumps of new infections through indirect contacts (e.g. low risk contact), combined with the presence of large number of susceptible herds in the area have resulted in larger epidemics size in ISP than DTU-DADS, when the index herd was cattle located in high density cattle area.

ISP tended to show larger variation and more extreme situations than the DTU-DADS (Table 1 and Figure 2). It is actually not completely clear why ISP creates larger variability and extreme situations than DTU-DADS. Nevertheless, the way disease spread is modelled might explain the larger variability predicted by ISP.

From this study, it was not possible to judge which way of modelling disease spread is the correct one. A way to get closer to the answer, would most likely be to compare models’ output to actual outbreak data, and then use the method that best explain the data. Recent outbreak data is not available in Denmark, given that the last outbreak was in 1982 [25]. Furthermore, it is actually unknown whether the models would have similar trends, as observed in the current study, had this exercise been conducted on data from another region. This is because the structure of the herds, the movement and contact patterns and intensity between herds in that region would also affect disease spread.

The models agreed that zone depopulation and suppressive vaccination are cheaper than the basic scenario. When depopulation and vaccination were compared, DTU-DADS was indifferent to the choices, while ISP estimated the costs, size and duration of suppressive vaccination to be smaller. This would indicate that the choice of control strategy might differ depending on the chosen model. However, from a practical point of view, the absolute differences were small, and given the large variation in the results, the final decision on strategy will most likely be based on other issues as well, such as practical, political, ethical and social effects of the epidemic. In this exercise, pre-emptive depopulation and suppressive vaccination were chosen to be implemented 14 day following the detection of the first infected herd. Following consultation with the National Veterinary Authorities, this timeframe seems reasonable before suppressive vaccination can be started. Despite that the two models did not fully agree on the chosen control strategy using this scenario, they have actually agreed that depopulation following the detection of 10 infected herds was the optimal scenario to control FMD spread in Denmark [15].

From a practical point of view, the advantage of DTU-DADS is that the structure of the model can be changed easily as soon as new data or knowledge arises, e.g. by adding new modules, because the source code is available. An important advantage, DTU-DADS runs on free software, but it demands personnel trained in programming. On the other hand, ISP is not free and cannot be extended to include changes to the structure of the model by the user. However, the model demands much less programming skills and training than DTU-DADS. ISP ran in few hours on personal computers, which is faster than the DTU-DADS that required one day for some scenarios in this study. Nonetheless, DTU-DADS can be run on a server, and hence can practically be very fast, because many scenarios can run at the same time. The cost-benefit analysis has been integrated within the DTU-DADS, which means that after the end of the model run, all necessary outputs can be obtained and only statistical analysis is still to be carried out. On the other hand, cost-benefit analysis on ISP outputs was carried out separately following the model run. Finally, DTU-DADS (in its current version) does not include elements of airborne spread, while ISP does. Important to mention, for the current exercise, spread of infection through airborne was not modelled, in order to keep the models as close to each other as possible. In a country where detailed herd, movement and contact data is available, ISP and DTU-DADS can both be useful, as they can represent the spread mechanism in details. This allows identifying risky contacts, which can be helpful to the veterinary authorities, while they are setting the preparedness and contingency plans.

The spread of FMD was compared using hypothetical data in 3 simulation models: ISP, North American Animal Disease Spread Model (NAADSM) and the Australian model (AusSpread) [17]. They found that the predicted number of infected premises and temporal and spatial spread predicted by the three models differed significantly, but the absolute differences were small and from a practical perspective would have resulted in a similar management decision being adopted. In a follow up study [18] and using actual population data, it was found that the predicted outcomes were also statistically significantly different between the different models, but the absolute results of ISP and AusSpread were clearly close compared to the results of the NAADSM, using the standard EU control measures [18]. In the current study, the results also showed frequently a statistically significant difference in the predicted outcomes of the 2 models, with small absolute differences as well.

The current study provided insight into the differences between the models and discussed how those differences could have influenced models’ predictions. Moreover, the current study estimated the financial impact of the epidemics, which is important, because significant epidemiological differences between the models could be financially indifferent or vice-versa. It is important to mention that efforts have been made to include the NAADSM in the comparison. Nonetheless, it was not possible to run the scenarios in the setup defined in the study using the available version of NAADSM when the study was performed, and thus the model was excluded. The restricted access to the source code of ISP has limited our capacity of investigating the effect of differences between the models on their predictions. Thus future research should have unlimited access to models’ code, and should focus on investigating, which method of modeling disease spread between herds would best represent reality. This can be done either by comparing the predicted outputs to outbreak data, or to kernel models that are estimated based on outbreak data [8], [9]. Furthermore, the larger variability that is predicted by ISP, compared to the DTU-DADS, should be further investigated.
